# YWHAH, a member of 14-3-3 family proteins, and PSME2, the proteasome activator subunit 2, are key host factors of Japanese encephalitis virus infection

**DOI:** 10.1186/s12920-023-01589-6

**Published:** 2023-07-10

**Authors:** Chaoyue Liu, Yanhong Yang, Qianqian Li, Weimin Hu, Jinxia Chang, Rong Chen, Hong Zhu, Mingfei Xu

**Affiliations:** Institute of Basic Medicine and Forensic Medicine, North Sichuan Medical College, Nanchong, 637000 China

**Keywords:** Japanese encephalitis virus, Host response, Differential Expression, Bioinformatics

## Abstract

**Background:**

Host response to virus infection is key to the effective control and eventual elimination of viruses or infected cells; however, the underlying mechanism of Japanese encephalitis virus (JEV) infection remains unclear.

**Methods:**

In the present study, short time-series expression was analyzed by R software to obtain two groups of differentially expressed genes (DEGs) [upregulated/downregulated] during the entire process of JEV infection based on the data in the Gene Expression Omnibus database. GO enrichment and KEGG pathway, protein interactions and hub genes selection were analyzed by DAVID, STRING and Cytoscape respectively. Interactions of the JEV and host proteins, and the microRNAs that target Tyrosine 3-monooxygenase/tryptophan 5-monooxygenase activating protein Eta (YWHAH) and Proteasome activator subunit 2(PSME2) were predicted by P-hipster and ENCORI, respectively. Expression levels of YWHAH and PSME2 were analyzed using the HPA database and RT-qPCR assay.

**Results:**

Two groups of continuously changed DEGs during entire process of JEV infection were obtained. Continuously upregulated cluster was mainly related to regulation of transcription, immune response and inflammatory response; and the continuous downregulated group mainly including intracellular protein transport and signal transduction, several proteolysis pathways. As targets of several microRNAs, the downregulated-YWHAH and the upregulated-PSME2 were related to host and JEV proteins to affect several pathways after JEV infection.

**Conclusions:**

YWHAH and PSME2 are key host factors of JEV infection based on their continuously differentially expressed pattern, interactions with multiple JEV proteins, and as members of the hub genes. Our results provide valuable information for further studies on the interactions between viruses and host.

**Supplementary Information:**

The online version contains supplementary material available at 10.1186/s12920-023-01589-6.

## Background

Japanese encephalitis (JE) is an acute central nervous system inflammatory disease that is caused by JEV [1]. According to WHO data, there are about 65,000 cases of JE worldwide every year, and it has become an important social public health event. So far, there is no effective antiviral drug and is partly due to an incomplete understanding of the mechanisms of JEV infection [2]. JEV is a small, enveloped, positive-strand RNA virus belonging to the family Flaviviridae, and it has one open reading frame (ORF) that encodes a single polyprotein that is cleaved into three structural proteins, namely, nucleocapsid (C), membrane (M), and envelope protein (E), and seven non-structural proteins (NS1, NS2A, NS2B, NS3, NS4A, NS4B, and NS5). Viral proteins interact with various host factors to complete its life cycle [3].

Host response to virus infection is key to the effective control and eventual elimination of invasive pathogens or infected cells. Virus infections can affect the RNA transcription of host cells in different ways, i.e., transcription may be downregulated on a large scale or mixed, with some upregulated and others downregulated. These changes in host gene expression may be a cellular antiviral response, a virus-induced response to promote self-replication and transmission, or a non-specific response that either promotes or prevents viral infection. Defining the transcriptional regulation of host genes during viral infection can be used as a tool to elucidate the mechanism of host-virus interactions and to establish its molecular pathogenesis. A previous study described major differences in gene expression profiles between mock-infected and JEV-infected microglia over time. However, the analyses focused on the middle and late stages of infection for only few differential genes were screened in the early stage of infection [4]. The present study, we utilize the same dataset (GSE57330) which was used in the referred study, focusing on the continuously upregulated or downregulated genes during the complete course of JEV infection to find more key host factors. Those DEGs continuously changed with the course of viral infection, so they can be used to assist the infection diagnosis through the expression level, help treatment by speculating infection stage, in addition to be used as a drug target based on their effects on viral replication. In fact, this type of research is very limited in virus research, but very extensive in tumor research, and a large number of studies have found that many continuous DEGs have strong ability in diagnostic and prognosis predictive. Fortunately, two important molecules YWHAH and PSME2 were obtained. YWHAH belongs to the 14–3-3 protein family and mediates signal transduction by binding to phosphoserine-containing proteins [5]. PSME2 is a member of the PA28 family and is also known as 11S regulator complex subunit beta (REG-beta) [6]. We hope our results would improve our understanding of the molecular events involved in disease progression, viral persistence, and related complex biological processes of host response.

## Materials and methods

### Databases

#### GEO (Gene Expression Omnibus)

**Fig. 1:**
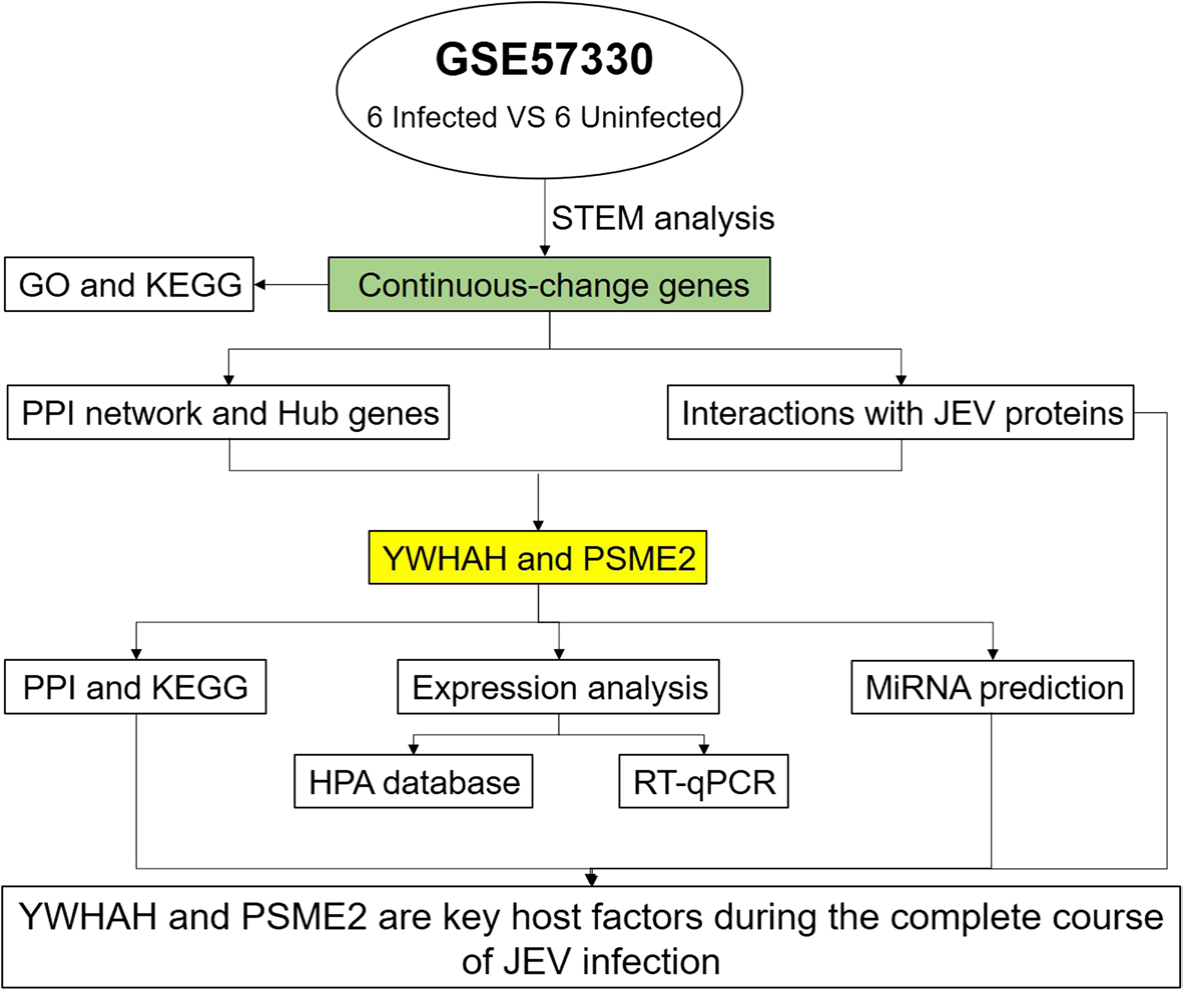
Flow chart of the entire analysis. STEM, short time-series expression miner

GEO is an international genetic database that is publicly accessible and freely available to researchers [7]. In our study, GSE57330 based on the GPL15207 platform was downloaded from GEO, which included six JEV-infected samples (human microglial cell line CHME3 cells grown on six-wells infected with an MOI of 5 pfu/cell of JEV P20778 strain at 6 h, 24 h and 48 h) and corresponding six JEV-uninfected samples. The ratio of expression values between infected group and uninfected group at the same time was used to represent the expression level. The analysis process is shown in Fig. 1. All genes in the dataset were organized into different clusters based on expression patterns using the “Mfuzz” package in R 3.6.3. “Mfuzz” is the R package used to perform the expression pattern clustering analysis of transcriptomic data at different time points. It is able to identify potential time-series patterns of expression profiles, and to cluster genes with similar patterns to help us understand the dynamic patterns of genes and their function links. So it was used to analyze the dynamic trends of all genes throughout the course of JEV infection to select the consistently varied-genes as the infection got worse.

#### DAVID (The Database for Annotation, Visualization and Integrated Discovery)

DAVID (https://david.ncifcrf.gov/home.jsp) provides researchers with a comprehensive functional annotation tool to help understand the biological significance behind a large number of genes [8]. In this study, we conducted Gene Ontology (GO) and Kyoto Encyclopedia of Genes and Genomes (KEGG) pathway enrichment analyses [9] of continuously up/downregulated genes that were isolated from DAVID, and the results were visualized with R 3.6.3.

#### STRING

STRING (https://cn.string-db.org/) is a database of known and predicted protein–protein interactions, currently covering 24,584,628 proteins from 5,090 organisms [10]. The interactions among the continuously up/downregulated genes were analyzed by STRING.

#### Cytoscape

Cytoscape is an open source software platform for visualizing complicated networks and integrating these with any type of attribute data [11]. A network construct of continuously up/downregulated genes was performed by Cytoscape, and hub genes were elected with the help of cytoHubba plate.

#### P-hipster (Pathogen-host interactome prediction using structure similarity)

P-hipster is an algorithm for inferring interactions between pathogens and human proteins using sequence and structural information [12]. The interactions of JEV proteins and host proteins were predicted by P-hipster.

#### HPA (Human Protein Atlas)

HPA (https://www.proteinatlas.org/) is a program that was initiated in 2003 that draws all human protein maps of cells, tissues, and organs by integrating various histological techniques. This resource is open access, allowing scientists in the academic and industrial community to utilize data to explore the human proteome [13]. The expression levels of YWHAH and PSME2 in tissues and cells were analyzed using the HPA database.

#### ENCORI (The Encyclopedia of RNA Interactomes)

ENCORI is an open-source platform for studying miRNA-ncRNA, miRNA-mRNA, ncRNA-RNA, RNA-RNA, RBP-ncRNA, and RBP-mRNA interactions from CLIP-seq, degradome-seq, and RNA-RNA interactome data and mainly focuses on the interactions of miRNAs and target molecules [14]. We predicted the microRNAs that target YWHAH and PSME2 using ENCORI.

### RT-qPCR

Human astroblastoma cells U87 cells were purchased from Procell Life Technology Co., Ltd., and cultured in the minimum essential medium (MEM) containing non-essential amino acids (NEAA) and 10% fetal bovine serum (FBS) at 37℃, 5% CO2. U87 cells were infected with an MOI of 5 pfu/cell of JEV (clone SCYA201201-86) at different time points post-infection (6, 24, and 48 h), and uninfected-U87 cells were used as negative control. Total RNA was extracted using TRNzol (Tiangen, DP424), followed by reverse-transcribed to cDNA using PrimeScript™ RT reagent Kit (Perfect Real Time) (Takara, RR037A). Subsequently, qPCR was performed in a 20 μL-reaction volume using TB Green® Premix Ex Taq™ (Tli RNaseH Plus) (Takara, RR420A) with specific primers (Table 1). With β-actin as reference gene, the relative transcription level of the PSME2 and YWHAH were calculated by the 2^−ΔΔCt^ method. The RNA transcript level of JEV NS1 was used to confirm the virus infection.

**Table 1:** Primers for qPCR

Gene name	Sequence (5’-3’)	Length(bp)
B-actin	Forward: CTTCGCGGGCGACGAT Reverse: CCACATAGGAATCCTTCTGACC	104
JEV NS1	Forward: GCGAAGATCGTCCACAAAGC Reverse: CACACTGAGGTCCACTGCAT	138
YWHAH	Forward: TGCTGGACTGATGGTTGCTT Reverse: TCAACTGTAGCCTGGTTGGC	163
PSME2	Forward: AATCTTTTCCAGGAGGCTGAGG Reverse: GGGAAGTCAAGTCAGCCACA	112

### Ethical approval statement

The Academic Committee of the North Sichuan Medical College agreed to our research protocols. All information for analysis was obtained from certified online sources, approval and informed consent from the local ethics committee were not required.

### Statistical analysis

The short time-series expression analyses and visualization were performed by R 3.6.3 Mfuzz package. Other software used included DAVID, STRING, Cytoscape, P-hipster, HPA, ENCORI. Statistical analysis of RT-qPCR was performed and plotted by Graphpad prism 6.0 software. Student’s t-test and analysis were used to evaluate the significant differences, and *P* value < 0.05 was defined as statistically significant.

## Results

### Multiple genes were defined as JEV-infected development characteristic genes

**Fig. 2:**
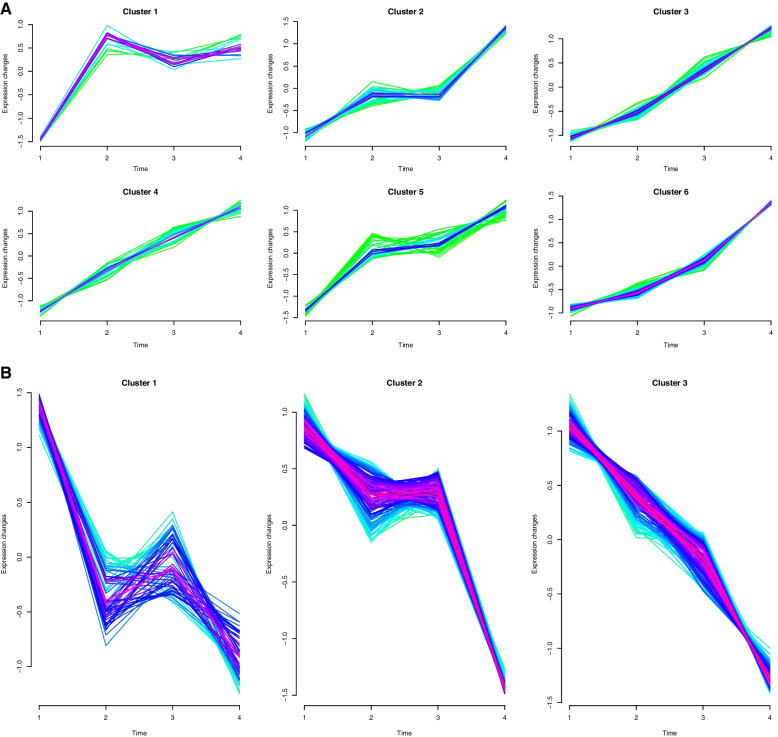
Four continuously changed-clusters were obtained during the entire course of JEV infection. Expression level of a gene was represented by the ratio of expression values between infected and uninfected group. Expression patterns of all genes in the dataset based on the expression levels of different time points were organized into different clusters using the “Mfuzz” package in R 3.6.3. **A** Upregulated clusters; **B** Downregulated clusters. The clusters in the red box represent the continuously changing clusters

One cluster of continuously upregulated genes and one cluster of continuously downregulated genes were screened by multiple short time-series expression analyses. Short time series gene expression experiments are widely used to study a range of biological processes such as the cell cycle, development, and immune response to help us understand the dynamic patterns of genes and their function links. We applied this method to analyze the dynamics of host genes during the whole process of JEV infection, and focused on genes that are consistently upregulated or downregulated. But in the clustering process, the trend of each cluster is difficult to meet the requirement at once due to the large number of genes. Therefore, after completing one analysis, the roughly qualified cluster will be selected for the second round of analysis, and through multiple rounds, the qualified cluster can be obtained. Approximately 115 genes were in the continuously upregulated cluster, and 147 genes were in the continuously downregulated cluster (Fig. 2, Supplementary file—Gene lists of continuously changed).

### KEGG and GO enrichment analyses of the continuously changing genes during JEV infection

**Fig. 3:**
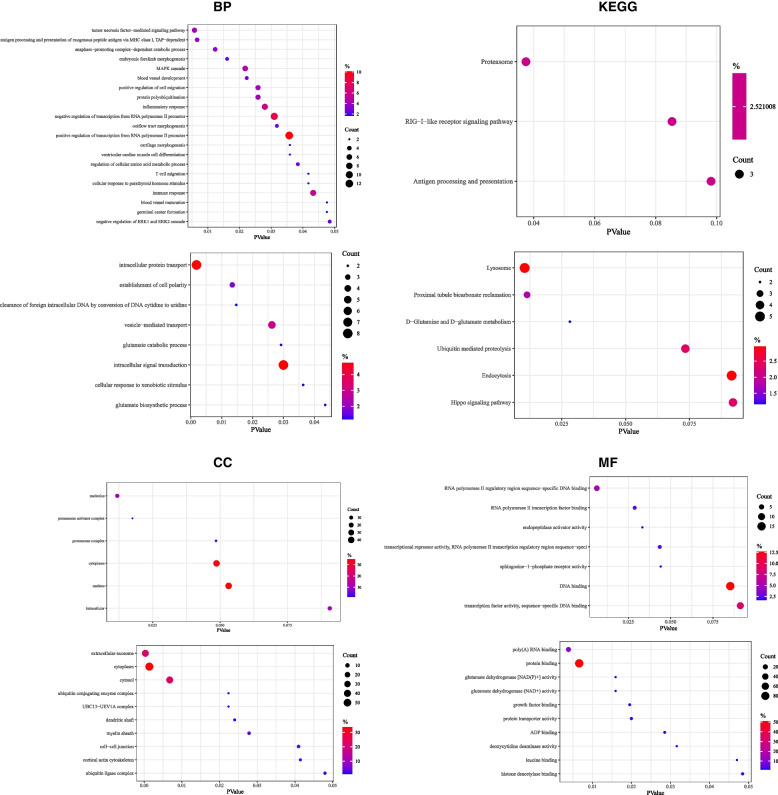
GO enrichment and KEGG pathway analyses of continuous-change genes during JEV infection. GO enrichment and KEGG pathway analyses of these genes in the continuously changed-clusters were performed with DAVID database, and the results were visualized with R 3.6.3. CC, Cellular component; BP, Biological process; MF, Molecular function. PValue, the probability value; Count, gene counts

GO is the abbreviation of Gene Ontology, which is used describe our knowledge of the biological domain with respect to three aspects: Molecular Function (MF), Cellular Component (CC), Biological Process (BP) [15]. KEGG pathway enrichment analysis is often applied to the functional annotation of DEGs to understand the related functions and pathways. It is well known that viral infection causes cell dysfunction and even triggers a series of associated cell processes that cause cell death [16]. We perform the KEGG and GO enrichment analyses to predict these continuously-altered genes involved cell functions and pathways. Figure 3 shows the most highly enriched GO and KEGG items.

In the continuously upregulated gene cluster, positive regulation of transcription from RNA polymerase II promoter, negative regulation of transcription from RNA polymerase II promoter, inflammatory response, immune response, MAPK cascade, tumor necrosis factor-mediated signaling pathway, positive regulation of cell migration, protein polyubiquitination, antigen processing, and presentation of exogenous peptide antigen via MHC class I (TAP-dependent) for BP category; cytoplasm, nucleus, nucleolus, and intracellular were the most highly enriched items in the CC category; in the MF category, enrichment was observed in terms of DNA binding, transcription factor activity, (sequence-specific DNA binding), and RNA polymerase II regulatory region sequence-specific DNA binding. In KEGG pathway, proteasome, RIG-I-like receptor signaling pathway, and antigen processing and presentation were screened.

In the continuously downregulated gene cluster, intracellular protein transport, intracellular signal transduction, vesicle-mediated transport, and establishment of cell polarity for BP category; and cytoplasm, cytosol, and extracellular exosome were the most highly enriched terms in the CC category; in the MF category, protein binding and poly (A) RNA binding were the most enriched terms. Additionally, six KEGG pathways were found, which included lysosome, endocytosis, ubiquitin-mediated proteolysis, Hippo signaling pathway, proximal tubule bicarbonate reclamation, and D-glutamine and D-glutamate metabolism.

It can be seen from above results, the involvement of the continuous changed-genes affect normal cell function in multiple aspects, which suggests them play an important role during JEV infection.

### Protein–Protein Interactions (PPI) network construction and hub gene analysis

**Fig. 4:**
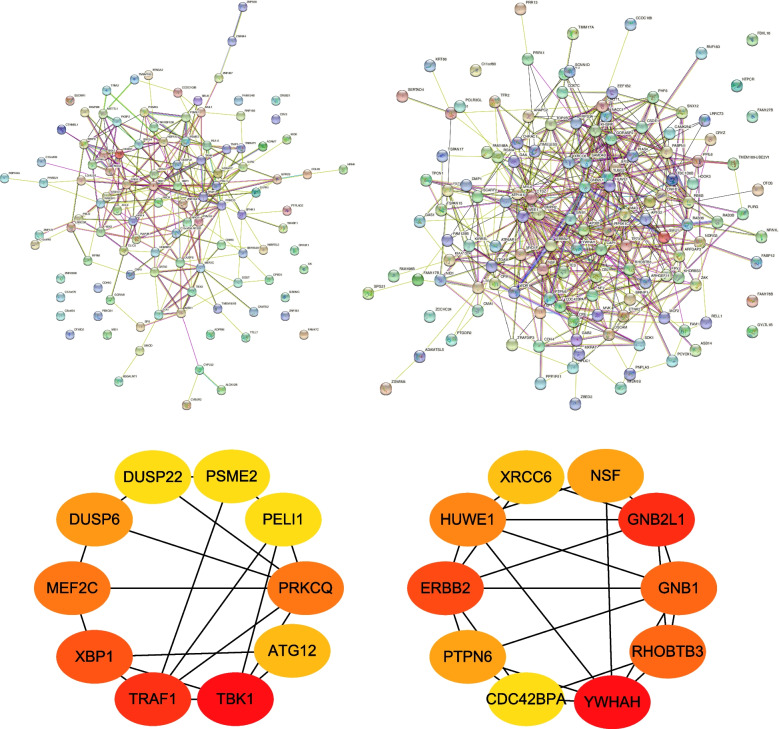
PPI network construction and hub gene analysis of these continuously changed DEGs. **A** PPI networks were constructed by STRING; **B** Hub gene were obtained by the “cytoHubba” plate of Cytoscape. Top 10 hub genes in the continuously up-regulated and down-regulated groups were displayed. The oval’s color is redder, the score is higher as a hub gene

We observed the connection among DEGs using STRING. Then we analyzed hub genes using cytoHubba in Cytoscape. In the upregulated group, NF-KB-Activating Kinase 1(TBK1), TNF Receptor-Associated Factor 1(TRAF1), X-Box-Binding Protein 1(XBP1), Protein Kinase C Theta Type (PRKCQ), Myocyte-Specific Enhancer Factor 2C(MEF2C), Dual Specificity Phosphatase 6 (DUSP6), Autophagy Related 12(ATG12), Pellino E3 Ubiquitin Protein Ligase 1(PELI1), PSME2, and Dual Specificity Phosphatase 22(DUSP22) were the top 10 genes, whereas in the continuously downregulated cluster, YWHAH, Receptor For Activated C Kinase 1(GNB2L1), Erb-B2 Receptor Tyrosine Kinase 2(ERBB2), Rho Related BTB Domain Containing 3(RHOBTB3), G Protein Subunit Beta 1(GNB1), HECT, UBA And WWE Domain Containing E3 Ubiquitin Protein Ligase 1(HUWE1), N-Ethylmaleimide Sensitive Factor, Vesicle Fusing ATPase (NSF), Protein Tyrosine Phosphatase Non-Receptor Type 6(PTPN6), X-Ray Repair Cross Complementing 6(XRCC6) and CDC42 Binding Protein Kinase Alpha (CDC42BPA) were on the top of the list (Fig. 4).

### YWHAH and PSME2 interact with multiple JEV proteins

**Fig. 5:**
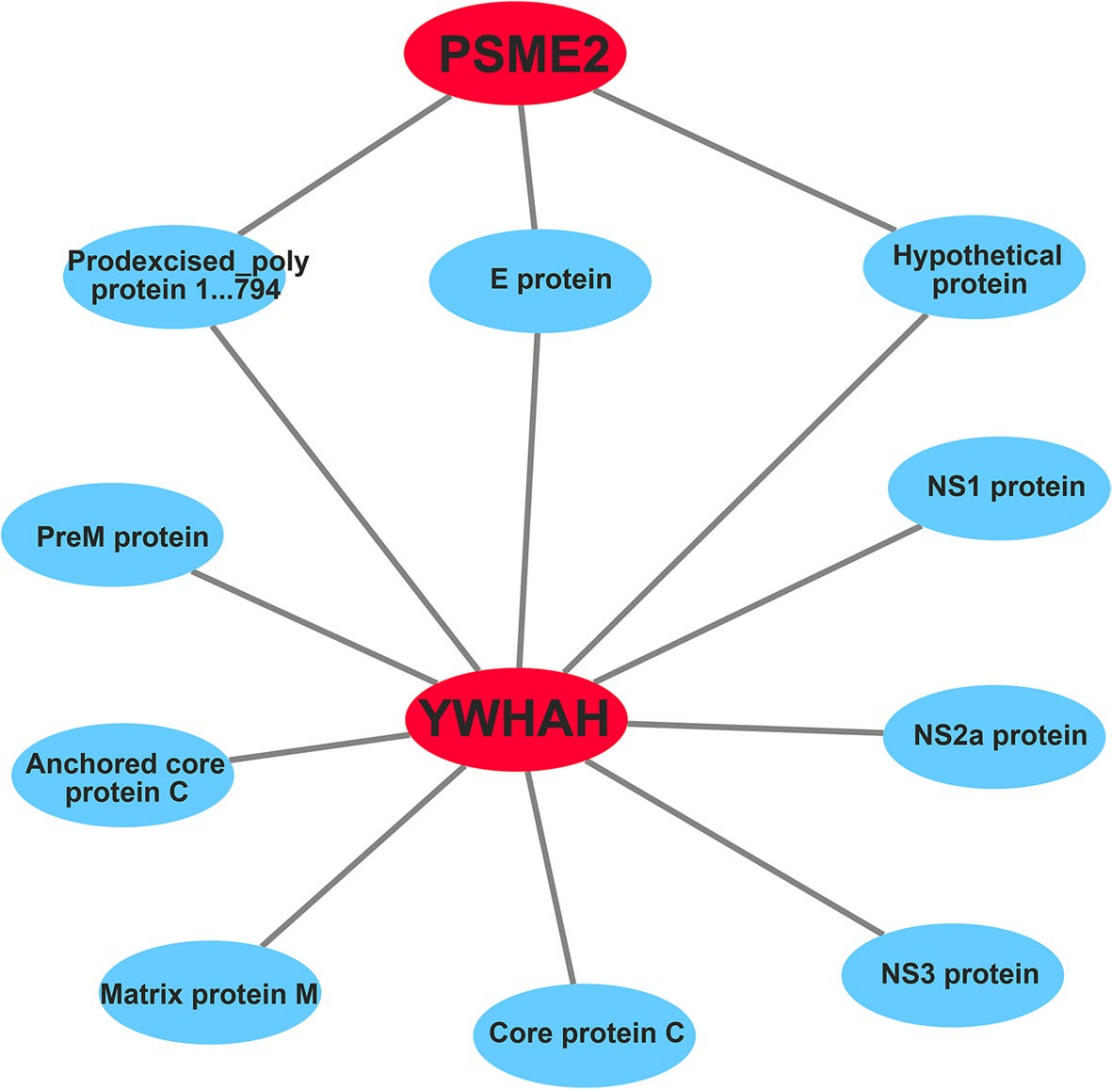
Two hub genes (YWHAH and PSME2) interact with JEV proteins. Interactions with JEV proteins of all the continuously changed genes were predicted by P-hipster database, and results indicated that only YWHAH and PSME2 belonging to hub genes interact with several JEV proteins

To investigate the role of these continuously changed genes (115 upregulated and 147 down regulated) in JEV infection, we analyzed the interactions with JEV proteins. The results showed only YWHAH and PSME2 interact with JEV proteins. The two proteins are in the hub gene networks, which suggests that these have important roles in JEV infection (Fig. 5).

### Expression of YWHAH and PSME2 in tissues and cells

**Fig. 6:**
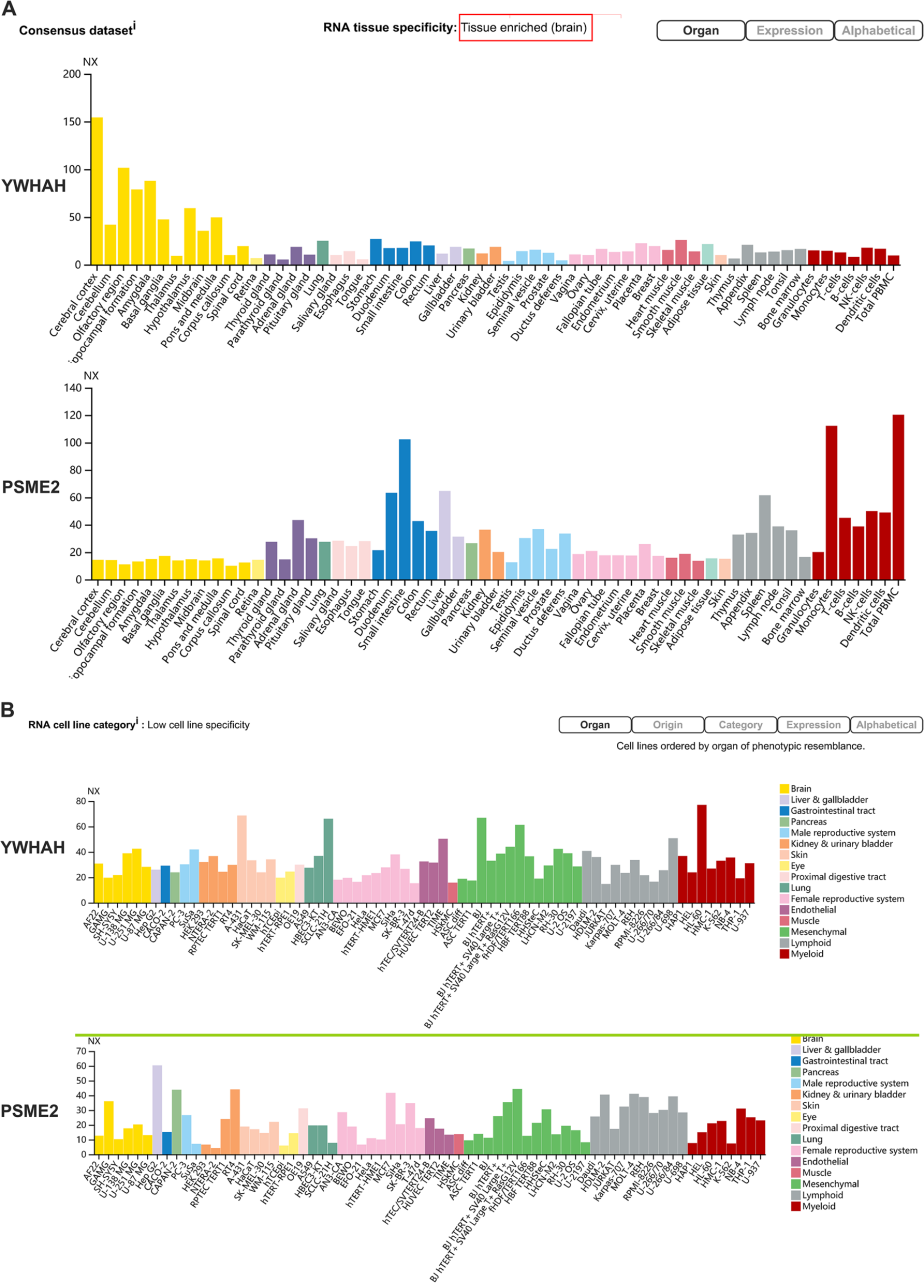
Expression analyses of YWHAH and PSME2 in tissues and cells. Expression levels of YWHAH and PSME2 in tissues and cells were analyzed using the HPA database. Expression of YWHAH is high in normal brain tissue and brain-related cell lines, while PSME2 Shows an opposite pattern. **A** Tissues; **B** Cells

**Fig. 7:**
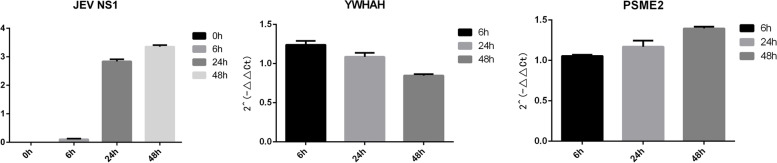
Verification of the expression patterns of YWHAH and PSME2 in U87. Expression of NS1, PSME2 and YWHAH in JEV-infected U87 cells at different time points post-infection (6, 24, and 48 h) were assessed by RT-qPCR. Expression level of NS1 as a marker of infection process. With β-actin as reference gene, the relative transcription levels of the YWHAH and PSME2 were calculated using the 2.^−ΔΔCt^method. With the progress of infection, YWHAH and PSME2 showed a pattern of continuous down-regulation and continuous up-regulation, respectively. **, *P* < 0.01; ***, *P* < 0.001

The expression levels of YWHAH and PSME2 in different tissues were analyzed using the HPA database. As Fig. 6A shown, YWHAH was specifically highly expressed in normal brain tissue, but not in other organs and tissues; PSME2 expression was extremely low in brain and relatively higher in total PMBC. In order to select a suitable cell model for follow-up mechanism research of JEV infection, the expression of YWHAH and PSME2 in different cell lines were analyzed. Expression of YWHAH was highest in U251 and U138, followed by U87, and PSME2 expression was extremely low in U87 and SH-SY5Y (Fig. 6B). The continuous change-regulation of YWHAH and PSME2 suggested that both play important roles in the pathogenesis of JEV. We performed RT-qPCR to verify the expression of YWHAH and PSME2 in U87 at different time points (6 h, 24 h, 48 h) after JEV infection (Fig. 7). The continuous downward trend of YWHAH and the continuous upward trend of PMSE2 were concordant with the above analyses.

### Analyses of PPI and KEGG and microRNA prediction of YWHAH and PSME2

**Fig. 8:**
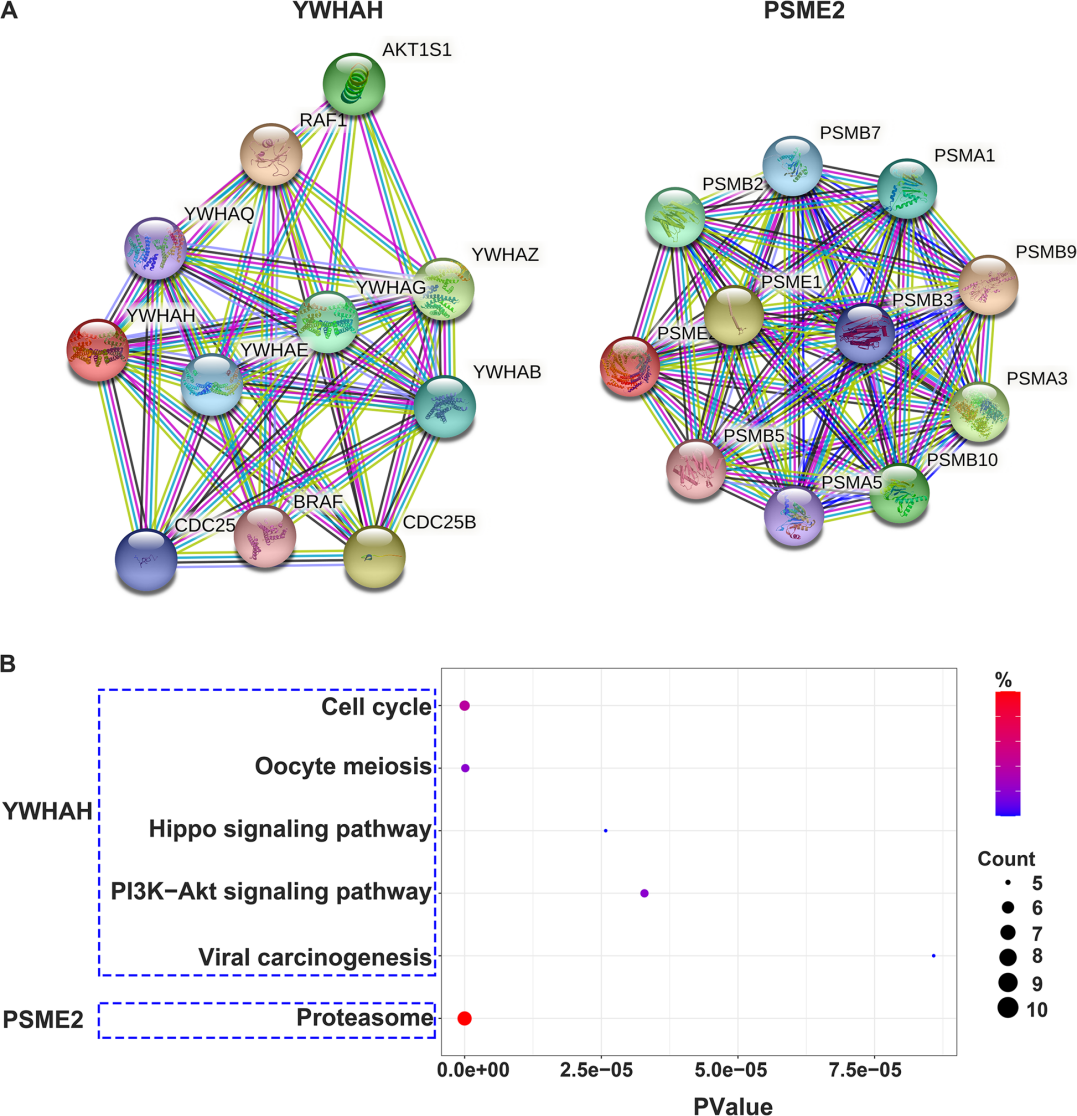
PPI network and KEGG analyses of proteins closely related to YWHAH and PSME2. **A** With the help of STRING database, proteins closely related to YWHAH and PSME2 were queried and PPI networks were constructed; **B** KEGG Pathway enrichment analyses of these closely related proteins were performed by using DAVID database, and the results were visualized with R 3.6.3. PValue, the probability value; Count, gene counts

**Fig. 9:**
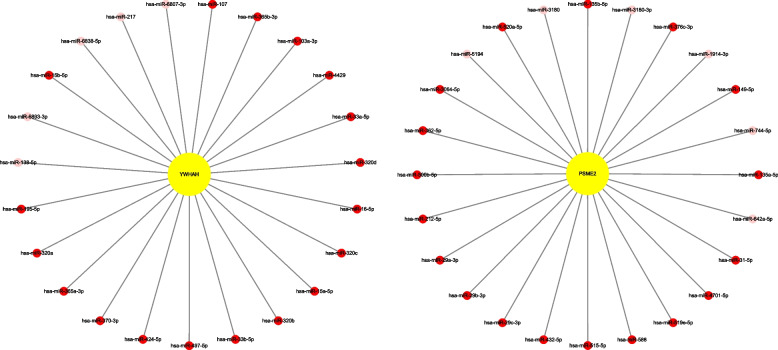
Predictions of microRNAs targeting YWHAH and PSME2. MicroRNAs that target YWHAH and PSME2 were predicted by using ENCORI database, and the interaction maps of these predicted miRNAs with the target gene PSME2 / YWHAH were plotted using Cytoscape software. The redder the color, the higher the credibility

To investigate the potential roles of YWHAH and PSME2 in JEV infection, we analyzed the closely interacting proteins of YWHAH and PSME2 and found that these enriched in the functional categories of cell cycle, PI3K-Akt signaling pathway, oocyte meiosis, viral carcinogenesis, Hippo signaling pathway for YWHAH and proteasome for PSME2 (Fig. 8). Finally, we predicted the microRNAs that target YWHAH and PSME2 using ENCORI (Fig. 9).

## Discussion

Japanese encephalitis B is mainly prevalent in Asia–Pacific region and is caused by JEV of the Flavivirus [17]. To understand the pathogenic mechanism of JEV, previous studies focused on DEGs at a certain time point after infection. Viral infection involves many steps; thus, focus on a certain time point may not be sufficient. In addition, studies have often focused on common DEGs; however, viral infection takes a certain time to cause significant changes in host gene expression, resulting in very few DEGs screened in the early stage of infection. Naturally, the number of common DEGs is also greatly reduced, and some important molecules involved in infection will also be missed. Therefore, in this study, we screened 115 and 147 genes that were continuously upregulated and downregulated, respectively, during infection. Then GO enrichment and KEGG pathway analyses were performed using DAVID. The results showed that these upregulated genes were mainly involved in transcriptional regulation (positive/negative), immune response, inflammatory response, and proteasome pathway. However, continuously downregulated genes were mainly involved in intracellular protein transport and signal transduction, protein binding, lysosome, endocytosis, ubiquitin-mediated proteolysis, and the Hippo signaling pathway. In addition, as infection progresses, the immune response and inflammatory response continue to increase. However, many important aspects related to protein physiological function, including transport, binding and signal transduction, protein degradation-related endocytosis, and lysosome and ubiquitin-mediated proteolysis are inhibited. This may be the route by which JEV completes replication and proliferation by inhibiting the function of host antiviral proteins and inducing the degradation of newly synthesized viral proteins or it may be the way by which host cells resist JEV by inhibiting the synthesis and assembly of JEV viral proteins, and then binding to the proteasome pathway to promote the degradation of newly synthesized viral proteins. More research is needed to determine whether these constantly changing genes are good for JEV or the host cells. In addition, we found that genes related to the Hippo signaling pathway were continuously downregulated during JEV infection. Hippo signal transduction is a highly evolutionarily conservative pathway that can control organ size by regulating cell proliferation, apoptosis, and stem cell self-renewal and plays an important role in cancer occurrence, tissue regeneration, and stem cell function regulation. Recent studies have found that the Hippo signaling pathway plays a key role in Zika virus replication and neuroinflammation [18], and studies on the specific role of the Hippo pathway in JEV are ongoing. Then, we constructed a molecular interaction network and screened hub genes in the two gene clusters. In the course of infection, host cell proteins also interact with viral proteins in addition to its own proteins. We analyzed the interaction of more than 150 proteins with the JEV virus protein and found that only the continuously upregulated PSME2 and the continuously downregulated YWHAH interacted with multiple JEV proteins. Interesting, both of them are belonged to be hub genes, which show the importance of the two genes. Therefore, qPCR was performed to verify the expression of PSME2 and YWHAH in U87 cells infected with JEV at different time points, which was concordant to the results of previous analysis.

YWHAH is one isoform of the 14–3-3 proteins, which have the highest tissue concentration in the brain [19]. YWHAH were reported to be related to many neurological diseases, including Parkinson’s disease [20], amyotrophic lateral sclerosis [21], Alzheimer’s disease [22], epilepsy, and Creutzfeldt-Jakob disease [22] and is also considered as a candidate gene for neurodegenerative diseases [23]. PSME2 was implicated in immunoproteasome assembly [24] and is required for efficient antigen processing [25], the most important disease of PSME2-related is immunodeficiency 12 [26]. Few studies have investigated the roles of these two proteins in viral infection, while the continuously changing expression levels combining with the interactions with JEV proteins indicated their importance in JEV infection. As we all known, JEV can cause neuroinflammation. Therefore, YWHAH as a protein closely related to neurological diseases, its roles in JEV infection should also be worthy of attention. In general, these two proteins have not been studied much in diseases, especially in viral infections. We hope our research could help promote them to get more attention in other viruses. Finally, we analyzed the proteins that closely interact with these two proteins and predicted the microRNAs that possibly regulate them to elucidate their roles and mechanisms, which may help guide further research.

This study has a number of limitations. First, all the data analyzed in our study were obtained from one cell type, and studies consisting of more cell types and brain tissue samples are required to validate our findings. Second, the expression of YWHAH and PSME2 in vivo were not verified in tissues and other cell types due to limitations in experimental conditions. Third, we did not conduct an in-depth analysis of the potential mechanisms of YWHAH and PSME2, and thus future studies are needed to explore the roles and mechanisms of YWHAH and PSME2 in JEV infection.

## Conclusions

In conclusion, we analyzed changes in host gene expression during the entire course of JEV infection. Our results indicated two group of genes that are continuously differentially expressed, and the upregulated cluster is mainly involved in transcriptional regulation, immune response, inflammatory response, and the proteasome pathway, whereas the continuously downregulated cluster is mainly related to intracellular protein transport, signal transduction and binding, protein degradation-related processes, and the Hippo signaling pathway. In addition, further analysis suggested that YWHAH and PSME2 are key host factors of JEV infection based on their continuously differentially expressed pattern, interactions with multiple viral proteins, and as members of the hub genes. Therefore, the above results may facilitate in elucidating the pathogenic mechanism of JEV infection.

## Supplementary Information

**Additional file 1.**

## Data Availability

The datasets used during the current study are publicly available from GEO (GSE57330).

## Abbreviations

DAVID: The Database for Annotation, Visualization and Integrated Discovery
P-hipster: Pathogen-host interactome prediction using structure similarity
KEGG: Kyoto Encyclopedia of Genes and Genomes
GO: Gene Ontology
ENCORI: Encyclopedia of RNA Interactomes
GEO: Gene Expression Omnibus
MHC: Major histocompatibility complex
ORF: Open reading frame
MAPK: Mitogen-activated protein kinase
BP: Biological process
HPA: Human Protein Atlas
MF: Molecular function
DEGs: Differentially expressed genes
JE: Japanese encephalitis
MOI: Multiplicity of Infection
CC: Cellular component
RIG-I-like: Retinoic acid-inducible gene I
JEV: Japanese encephalitis virus
